# Innate sensing of microbial products promotes wound-induced skin cancer

**DOI:** 10.1038/ncomms6932

**Published:** 2015-01-09

**Authors:** Esther Hoste, Esther N. Arwert, Rohit Lal, Andrew P. South, Julio C. Salas-Alanis, Dedee F. Murrell, Giacomo Donati, Fiona M. Watt

**Affiliations:** 1 Cancer Research UK Cambridge Research institute, Li Ka Shing Centre, Robinson Way, Cambridge CB2 0RE, UK; 2 Centre for Stem Cells and Regenerative Medicine, King’s College London, 28th Floor, Tower Wing, Guy’s Campus, London SE1 9RT, UK; 3 Cancer Clinical Academic Group, Guy's and St Thomas' NHS Trust, Bermondsey Wing, Guy's Hospital, Great Maze Pond, London SE1 9RT, UK; 4 Division of Cancer Research, Ninewells Hospital and Medical School, University of Dundee, Dundee DD1 9SY, UK; 5 Department of Dermatology and Cutaneous Biology, Thomas Jefferson University, Philadelphia, Pennsylvania 19107, USA; 6 Basic Science Department, Medicine School, University of Monterrey, Nuevo Leon 64849, Mexico; 7 Department of Dermatology, St George Hospital, University of New South Wales, Sydney, New South Wales 2217, Australia

## Abstract

The association between tissue damage, chronic inflammation and cancer is well known. However, the underlying mechanisms are unclear. Here we characterize a mouse model in which constitutive epidermal extracellular-signal-regulated kinase-MAP-kinase signalling results in epidermal inflammation, and skin wounding induces tumours. We show that tumour incidence correlates with wound size and inflammatory infiltrate. Ablation of tumour necrosis factor receptor (TNFR)-1/-2, Myeloid Differentiation primary response gene 88 or Toll-like receptor (TLR)-5, the bacterial flagellin receptor, but not other innate immune sensors, in radiosensitive leukocytes protects against tumour formation. Antibiotic treatment inhibits, whereas injection of flagellin induces, tumours in a TLR-5-dependent manner. TLR-5 is also involved in chemical-induced skin carcinogenesis in wild-type mice. Leukocytic TLR-5 signalling mediates upregulation of the alarmin HMGB1 (High Mobility Group Box 1) in wound-induced papillomas. HMGB1 is elevated in tumours of patients with Recessive Dystrophic Epidermolysis Bullosa, a disease characterized by chronic skin damage. We conclude that in our experimental model the combination of bacteria, chronic inflammation and wounding cooperate to trigger skin cancer.

## Abstract (web summary)

Inflammation and wounding can promote cancer development, but the underlying mechanisms are unclear. Here Hoste *et al.* show that leukocytes sensing flagellin, a component of bacteria, play a key role in inducing skin cancer in the context of wounding and chronic inflammation.

## Graphical abstract

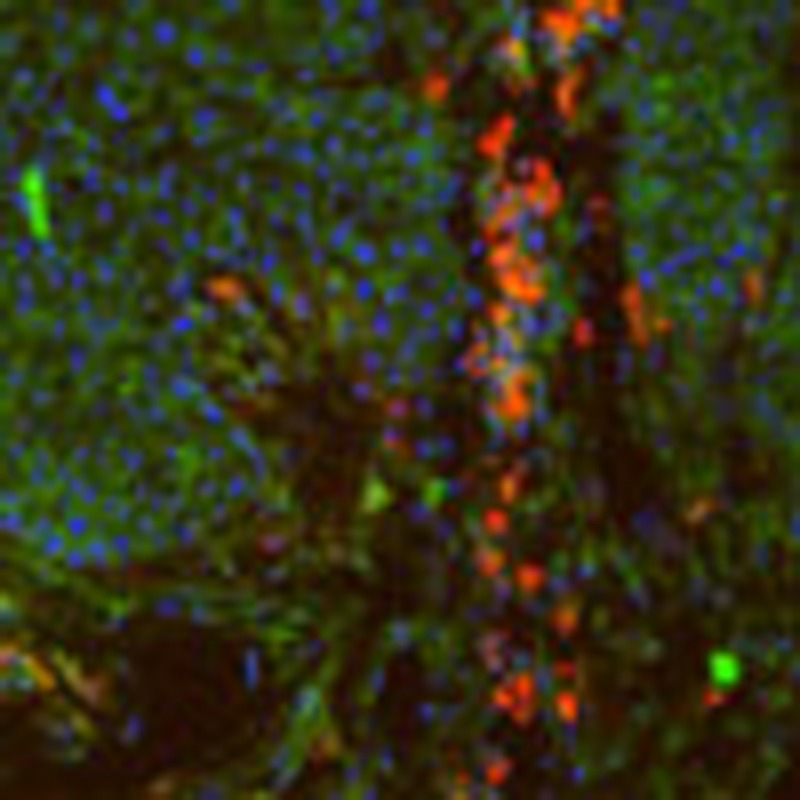

The association between skin wounding, inflammation and cancer is well established^1^,^2^. For example, Marjolin’s ulcers are aggressive squamous cell carcinomas (SCCs) that specifically develop on areas of previous skin trauma^3^. Keloid scarring is a consequence of aberrant wound healing and is also described as benign fibrotic tumour formation^4^. In addition, malignancies at wound sites are often overlooked in chronic ulcers in diabetic and elderly patients^5^. Another context in which the association between skin wounding and cancer is well established is Recessive Dystrophic Epidermolysis Bullosa (RDEB). This inherited skin blistering disease is characterized by repetitive cycles of wounding and repair and is linked with a high incidence of SCC formation. Almost 100% of RDEB patients will develop at least one SCC^6^. However, despite the clear link between skin damage and cancer, little is known about the underlying mechanisms.

We previously described a mouse model of wound-induced skin cancer that mimics key features of human hyperproliferative skin conditions. Wounded human skin and psoriatic lesions are characterized by misexpression of β1 integrin extracellular matrix receptors in the differentiating epidermal cell layers, and consequent upregulation of extracellular signal-regulated kinase-MAP-kinase signalling^7^. When this is modelled in transgenic mice by expression of constitutively active MAP-kinase kinase 1 under the control of the involucrin promoter (InvEE mice), there is chronic skin inflammation and epidermal hyperproliferation, and mice develop benign tumours (papillomas) on wounding^8^,^9^. We previously identified a pro-tumorigenic role for macrophages and peripheral γδT cells in this model^8^.

Here we set out to identify the molecular signalling events that underlie the link between chronic inflammation, tissue damage and skin cancer. We have found a previously unknown role for Toll-like receptor (TLR)-5, the receptor for bacterial flagellin, in skin tumour formation, both in the InvEE mouse model and in wild-type (WT) mice treated with chemical carcinogens. We further show that TLR-5 plays a role in upregulation of the alarmin HMGB1 (High Mobility Group Box 1) in wound-induced mouse tumours and demonstrate that HMGB1 is also elevated in tumours of RDEB patients.

## Results

### Effect of wound size and immune infiltrate on tumorigenesis

**Figure 1:**
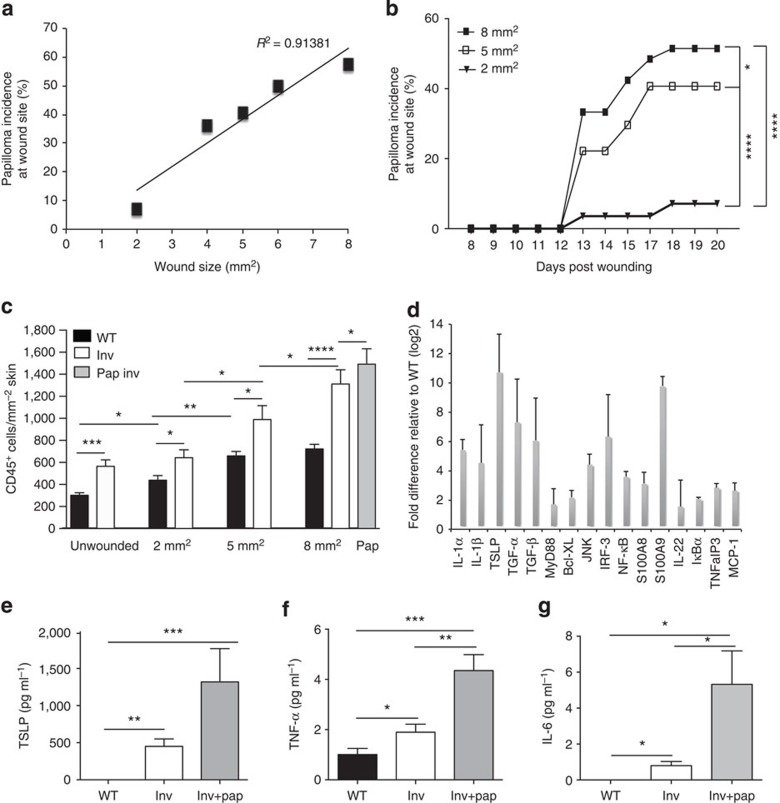
Pro-inflammatory signalling and wound-induced tumour formation in InvEE mice. (**a**) Correlation between wound diameter and papilloma incidence at wound site in InvEE mice 30 days post wounding (*n*>20 mice per condition). (**b**) Incidence of tumours in InvEE mice in 2 mm^2^ (*n*=28 mice), 5 mm^2^ (*n*=27 mice) or 8 mm^2^ (*n*=33 mice) wounds (*0.01<*P*<0.05; ****P<0.0001; one-way analysis of variance (ANOVA)). (**c**) Quantification of CD45^+^ leukocytes in different sized wounds at time of wound closure (*n*≥4 mice per condition, at least three microscopic fields were quantified per mouse; *0.01<*P*<0.05, **0.01<*P*<0.001, ***0.0001<*P*<0.001, ****P<0.0001; two-way ANOVA). (**d**) Quantitative PCR normalized to glyceraldehyde 3-phosphate dehydrogenase of NF-κB target gene mRNAs in 8-week-old InvEE epidermis relative to transgene negative littermates (log 2-fold upregulation relative to WT; *n*=3 mice per condition). Data are means±s.d. of biological and technical triplicates (*P*<0.0001 for each individual gene product; unpaired *t*-test). (**e**–**g**) Serum levels of TSLP (**e**), TNF-α (**f**) and IL-6 (**g**) in control, tumour-free and tumour-bearing InvEE mice, assessed by ELISA (*n*=4 mice per condition; *0.01<*P*<0.05, **0.01<*P*<0.001, ***0.0001<*P*<0.001; unpaired *t*-test). Data are means±s.e.m.; pap: papilloma.

To test whether wound size, wound closure rate or inflammatory response to wounding influenced tumour incidence, full-thickness skin wounds of different sizes (2, 4, 5, 6 and 8 mm^2^) were made on back skin of WT and InvEE (Inv) mice, and papilloma formation at the wound site was monitored. Wounds in InvEE and WT littermates healed at the same rate but only InvEE mice developed tumours (Supplementary Fig. 1a,b). Although onset of tumour formation was independent of wound size, there was a linear correlation between wound size and tumour incidence (*R*^2^=0.91381; Fig. 1a,b). Wound size and total immune cell infiltrate (CD45^+^ cells) were correlated in both WT and InvEE skin, but CD45^+^ cells were significantly more abundant in InvEE skin both before wounding^9^ and at the time of wound closure (Fig. 1c and Supplementary Fig. 1c,d). There were even more CD45^+^ cells in the tumour stroma than in newly closed 8 mm^2^ InvEE wounds (Fig. 1c). These results indicate that the degree of inflammation remaining once the acute response to injury has resolved correlates with the extent of the primary insult and subsequent tumour incidence.

As nuclear factor-κB (NF-κB) is an important mediator of inflammation-associated cancer^10^, we analysed expression of NF-κB target genes in InvEE and control epidermis. All 16 of the genes examined were significantly upregulated in InvEE epidermis relative to WT epidermis (*P*<0.0001 for each individual gene product; Fig. 1d). The effects were systemic, as levels of thymic stromal lymphopoietin (TSLP), tumour-necrosis factor (TNF)-α and interleukin (IL)-6 were elevated in serum of tumour-free InvEE mice and increased further in tumour-bearing animals (Fig. 1e–g).

### Tumour formation requires haematopoietic TNFR signalling

TNF-α is well known for its context-dependent pro- and anti-tumorigenic roles^11^ and downstream TNF-α signalling is mediated by TNFR-1 and TNFR-2. Mice deficient in both receptors are resistant to skin cancer induced by chemical carcinogens^12^. To examine whether wound-induced tumorigenesis was dependent on TNFR signalling specifically in leukocytes, sub-lethally irradiated InvEE mice were reconstituted with TNFR-1/-2^−/−^ (TNFR^−/−^) bone marrow (BM) and subsequently wounded. Successful engraftment was verified by Y chromosome-fluorescence *in situ* hybridization (Y-FISH) in spleens of reconstituted mice as previously described^8^ (Supplementary Fig. 2a).

**Figure 2:**
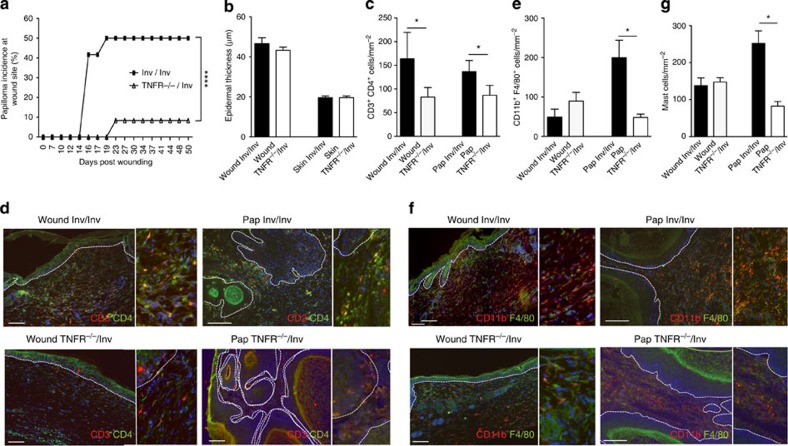
Role of TNFR signalling in wound-induced tumours. (**a**) Incidence of papillomas in TNFR-1/-2^−/−^ (TNFR^−/−^/Inv; *n*=12) and control (Inv/Inv; *n*=12) BM chimeras (****P<0.0001; Fisher’s exact test). (**b**) Epidermal thickness in healed and unwounded skin, defined as the distance from basal to upper granular layer and measured at ten sites per mouse (wound Inv/Inv: *n*=4; wound TNFR^−/−^/Inv: *n*=8; skin Inv/Inv: *n*=8; skin TNFR^−/−^/Inv: *n*=10). (**c**–**g**) Infiltration of different immune cell populations in wound beds and tumour stroma of post-wounded InvEE and TNFR^−/−^ chimeric skin (*0.01<*P*<0.05; unpaired *t*-test). (**d**,**f**) Histological sections were stained with antibodies to CD3 (red in **d**) and CD4 (green in **d**) or CD11b (red in **f**) and F4/80 (green in **f**) and double positive cells were quantified (**c**,**e**). Magnified views are represented on the right. (**g**) Mast cells were quantified following toluidine blue staining. (**c**,**e**,**g**) n≥3 mice per condition and ≥3 fields were quantified per section. Means±s.e.m. are shown. (**d**,**f**) Nuclei were stained with 4,6-diamidino-2-phenylindole (blue); dotted lines indicate basement membrane. Scale bars, 300 μm.

TNFR^−/−^ chimeric mice were highly resistant to wound-induced tumour formation (Fig. 2a). Only 8.3% of TNFR^−/−^ chimeric mice developed papillomas, compared with 50% of control chimeras, and time of tumour onset was delayed in TNFR^−/−^ chimeric mice (Fig. 2a). Although wounds closed more rapidly in TNFR^−/−^ chimeras (Supplementary Fig. 2b), in agreement with observations on TNFR-1^−/−^ mice^13^, the epidermis remained thickened and hyperproliferative, consistent with the ability of MEK1 to stimulate keratinocyte proliferation in the absence of other cell types^7^ (Fig. 2b). Serum levels of TNF-α were markedly reduced in tumour-free but not tumour-bearing TNFR^−/−^ chimeras (Supplementary Fig. 2c), consistent with MEK1 activation in epidermal tumour cells driving NF-κB activation^14^.

The tumour-protective effect of TNFR ablation in radiosensitive leukocytes correlated with changes in the skin immune cell infiltrate. CD4^+^ T cells were markedly reduced in wounds and papillomas of TNFR^−/−^ BM chimeras (Fig. 2c,d). When irradiated InvEE mice were reconstituted with BM from mice expressing enhanced green fluorescent protein under the control of the β-actin cytomegalovirus (CMV) promoter and subsequently wounded, both the wounds and wound-induced tumours were heavily infiltrated with F4/80^+^ macrophages (Supplementary Fig. 2d). Macrophage (F4/80^+^ CD11b^+^) and mast cell numbers were similar in healed wounds of TNFR^−/−^ and control chimeras but significantly reduced in tumour stroma of TNFR^−/−^ chimeras (Fig. 2e–g).

Epidermal γδ T cells infiltrated wounds of both TNFR^−/−^ and control chimeras to the same extent. They were never present within the tumour epithelium, but did accumulate in adjacent epidermis (Supplementary Fig. 2e), suggesting that the previously observed reduction in tumours on γδ T-cell ablation is an indirect effect of reduced macrophage recruitment^8^. TNFR ablation in the BM did not affect numbers of dendritic cells (CD207^+^ CD11c^+^), NK or NKT cells infiltrating wounds or tumours (Supplementary Fig. 2f–h). B cells (CD19^+^) were not detectable in unwounded skin or healed wound beds^15^ and there was no difference in the stromal B-cell content of TNFR^−/−^ and control chimeric tumours (Supplementary Fig. 2i).

We conclude that TNFR ablation in leukocytes protected mice from developing tumours. It also led to a selective reduction in CD4^+^ T cells in wounded skin and a reduction in several immune cell subsets in tumour stroma.

### MyD88 and TLR-5 signalling mediate tumour formation

**Figure 3:**
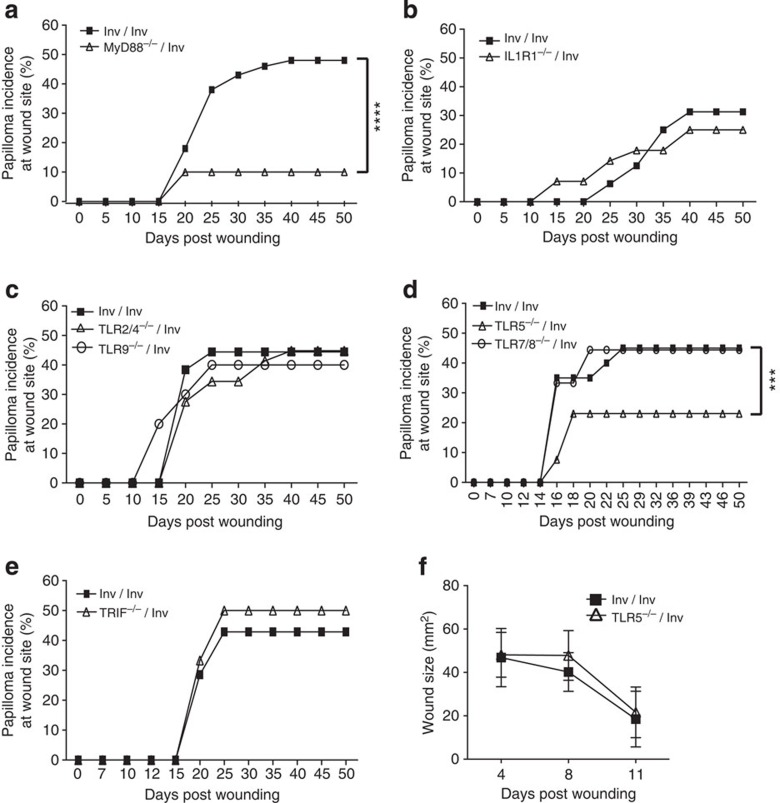
MyD88 and TLR-5 signalling on radiosensitive leukocytes is required for tumour formation. (**a**–**e**) Incidence of papillomas at wound site. (**a**) MyD88^−/−^ (MyD88^−/−^/Inv; *n*=20) and control BM chimeras (Inv/Inv; *n*=20; ****P<0.0001; Fisher’s exact test). (**b**) IL-1R1^−/−^ (IL-1R1^−/−^/Inv; *n*=28) and control BM chimeras (Inv/Inv; *n*=16; *P*=0.4312; Fisher’s exact test). (**c**) TLR-2/−4^−/−^ (TLR-2/-4^−/−^/Inv; *n*=29), TLR-9^−/−^ (TLR-9^−/−^/Inv; *n*=10) and control BM chimeras (Inv/Inv; *n*=30; *P*>0.05; one-way analysis of variance (ANOVA)). (**d**) TLR-7/-8^−/−^ (TLR-7/−8^−/−^/Inv; *n*=9), TLR-5^−/−^ (TLR5^−/−^/Inv; *n*=13; ***0.0001<*P*<0.001; one-way ANOVA) and control BM chimeras (Inv/Inv; *n*=20). (**e**) TRIF^−/−^ (TRIF^−/−^/Inv; *n*=12) and control BM chimeras (Inv/Inv; *n*=14; *P*>0.05; Fisher’s exact test). (**f**) Rate of wound healing in TLR5^−/−^ and control BM chimeras (*n*=12 per condition; *P*>0.05; unpaired *t*-test).

MyD88 (Myeloid Differentiation primary response gene 88) is a master regulator of innate signalling events as it is the key adaptor for most TLRs, IL-1R1 and IL-18R^16^,^17^,^18^. Loss of MyD88 prevents tumour formation in various tissues^19^,^20^,^21^,^22^. Given that MyD88 controls TNF-α production, we analysed the effect of reconstituting InvEE mice with MyD88^−/−^ radiosensitive leukocytes. BM chimeras lacking MyD88 in the haematopoietic compartment exhibited a striking protection against wound-induced tumour formation (Fig. 3a).

Although InvEE keratinocytes express elevated levels of IL-1α and administration of the IL-1 receptor antagonist Kineret decreases tumour formation^8^,^9^, no differences in wound-induced tumour formation were observed between IL-1R1^−/−^ BM and control chimeras (Fig. 3b). We therefore examined the effects of deleting TLRs. Replacement of the radiosensitive haematopoietic compartment with TLR-2/-4^−/−^ or TLR-9^−/−^ cells did not affect tumour formation (Fig. 3c), in contrast to the role of TLR-4 on haematopoietic and non-haematopoietic cells in chemically induced skin carcinogenesis^23^. InvEE mice reconstituted with TLR-7/-8^−/−^ BM exhibited accelerated wound closure (Supplementary Fig. 3a) but no difference in tumour incidence was observed (Fig. 3d). TLR-3 and TLR-4 can signal via TIR-domain-containing adapter-inducing interferon-β (TRIF), instead of MyD88. However, reconstitution with TRIF^−/−^ BM cells had no effect on papilloma formation (Fig. 3e).

Ablation of TLR-5 in radiosensitive leukocytes markedly reduced the number of tumours that developed on wounding (Fig. 3d). Wound closure rates were similar in TLR-5^−/−^ and control BM chimeras, suggesting that the dynamics of wound closure does not affect wound-induced tumour formation (Fig. 3f).

These findings reveal the significance of an innate MyD88-TLR-5-sensing axis specifically in BM-derived leukocytes that drives wound-induced tumour initiation.

### Bacterial products mediate tumour initiation in InvEE mice

**Figure 4:**
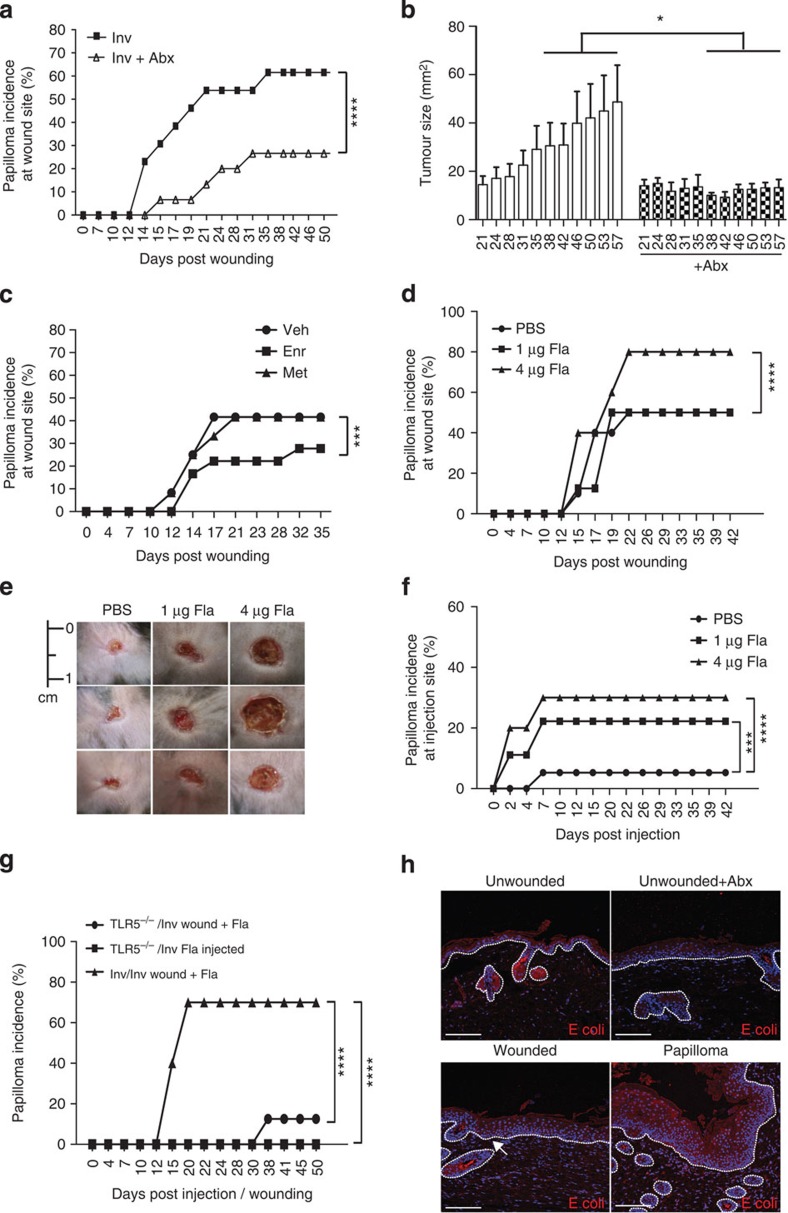
Microbial products promote tumour formation in InvEE mice. (**a**,**c**,**d**,**f**,**g**) Incidence of papilloma formation following wounding (**a**,**c**,**d**,**g**) or intradermal injection (**f**,**g**). (**a**) Mice were untreated (*n*=13) or treated with orally administered enrofloxacin (Enr) antibiotic (*n*=15) starting 8 days before wounding (*P*<0.0001; Fisher’s exact test). (**b**) Tumour growth in untreated (*n*=8) and oral antibiotic-treated (*n*=4) mice (*0.01<*P*<0.05; unpaired *t*-test). Data are means±s.e.m. (**c**) Mice were treated topically with vehicle (Veh; acetone; *n*=12), Enr (*n*=21) or methicillin (Met; *n*=12; ***0.0001<*P*<0.001; one-way analysis of variance (ANOVA)). (**d**) PBS (*n*=10), 1 μg flagellin (Fla; *n*=8) or 4 μg Fla (*n*=10) was applied in wound at time of wounding (****P<0.0001; one-way ANOVA). (**e**) Wounds treated with flagellin or PBS were photographed 7 days after wounding. (**f**) Papilloma incidence in mice that received intradermal injections of PBS (*n*=19), 1 μg Fla (*n*=8) or 4 μg Fla (*n*=10; ***0.0001<*P*<0.001; one-way ANOVA). (**g**) Papilloma incidence in TLR-5^−/−^ BM chimeras (*n*=8) and control (*n*=10) chimeras that were wounded or injected (*n*=10) with 4 μg Fla (***0.0001<*P*<0.001; one-way ANOVA). (**h**) Immunofluorescence labelling of InvEE skin with antibody to flagellated *E. coli* K12 strain. Wounded skin was collected 17 days after wounding. Unwounded skin was either untreated or treated (+Abx) for 8 days with oral Enr. Dotted line represents epidermal–dermal boundary. Scale bars, 300 μm.

As flagellin (Fla), the sole known TLR-5 ligand^24^, is the main protein constituent of bacterial flagella, we analysed whether lowering the microbial content of the skin would affect wound-induced tumour incidence. When mice were treated with the broad-spectrum antibiotic enrofloxacin (enr), either by administration in drinking water or topical application, the skin bacterial load was decreased (Supplementary Fig. 3c, d) and wound-induced tumour formation was greatly reduced (Fig. 4a,c). Tumour size in antibiotic-treated mice was greatly reduced (Fig. 4b), an effect that was previously observed in intestinal tumours^25^. No reduction in tumour initiation was observed when mice were topically treated with methicillin (met; Fig. 4c), a narrow-spectrum antibiotic that targets Gram-positive bacteria that are highly abundant on the skin, including the non-flagellated species *Staphylococcus aureus* and *Staphylococcus epidermidis*.

In mice topically treated with antibiotics, there was no significant reduction in faecal bacterial load, excluding involvement of the gut microbiome in wound-induced skin carcinogenesis (Supplementary Fig. 3e). Broad-spectrum antibiotic treatment resulted in a transient increase in wound closure in WT but not InvEE mice (Supplementary Fig. 3f).

In contrast to the tumour-suppressive effects of TLR-5 ablation and antibiotic treatment, topical application of flagellin to InvEE wounds increased tumour incidence in a dose-dependent manner (Fig. 4d) and delayed wound closure (Fig. 4e and Supplementary Fig. 3b). Strikingly, intradermal injection of flagellin was sufficient to induce small tumours in InvEE mice in the absence of wounding (Fig. 4f). WT mice never developed tumours after administration of flagellin to wounds or intradermal injection (data not shown). Flagellin did not induce tumours when injected into TLR-5^−/−^ BM chimeras. When flagellin was administered to wounds of TLR-5^−/−^ BM chimeras, tumour formation was greatly diminished compared with control chimeras treated with flagellin (Fig. 4g).

Flagellated bacterial strains are commensals on murine skin^26^. When we labelled unwounded InvEE skin with an antibody to the flagellated *Escherichia coli* (*E. coli*) strain K12, we observed strong immunoreactivity in hair follicles, sebaceous glands and cornified skin layers (Fig. 4h), in agreement with a previous report^26^. As expected, *E. coli* labelling was markedly reduced in antibiotic-treated skin. All epidermal layers stained positive for K12 *E. coli* in InvEE healed wound beds and papillomas (Fig. 4h).

Taken together, these data demonstrate that exposure to bacterial flagellin sensed by TLR-5 on radiosensitive leukocytes promotes tumour formation in InvEE mice.

### Role of TLR-5 signalling in carcinogen-induced tumours

**Figure 5:**
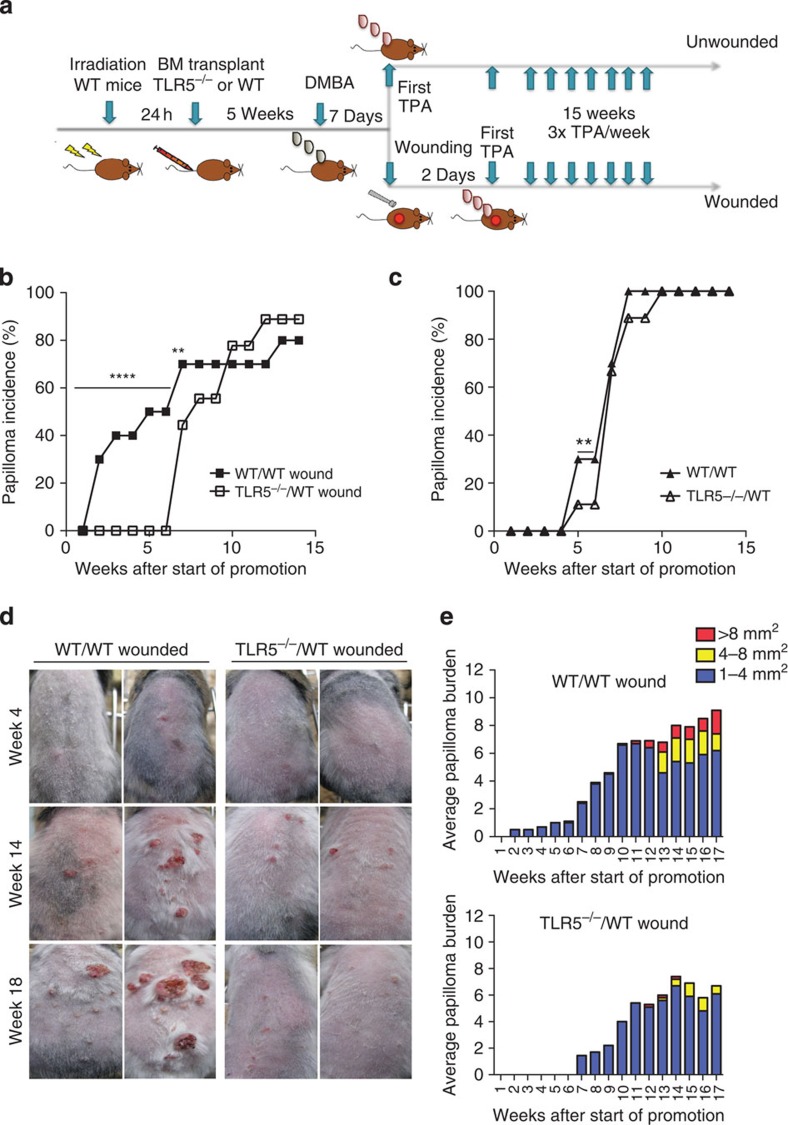
TLR-5 signalling in leukocytes promotes DMBA/TPA wound-induced tumorigenesis. (**a**) Schematic of DMBA/TPA wound-induced tumorigenesis protocol. (**b**,**c**) Incidence of papilloma formation in WT mice reconstituted with TLR-5^−/−^ (WT/TLR5^−/−^) or control (WT/WT) BM and treated with DMBA and TPA with (**b**) or without (**c**) wounding. (**b**) *n*=10 WT/WT mice; *n*=9 TLR5^−/−^/WT mice; ****P<0.0001; unpaired *t*-test. (**c**) *n*=10 WT/WT mice; *n*=9 TLR5^−/−^/WT chimeras; **0.01<*P*<0.001; unpaired *t*-test. (**d**) Back skin of representative wounded WT/WT and WT/TLR5^−/−^ mice, 4, 14 and 18 weeks after start of TPA treatment. (**e**) Average total number of tumours and tumour size measured weekly after first TPA treatment. *n*>8 mice per condition.

To validate our observations in a second experimental setting, we induced tumours in WT mice via the classic two-stage DMBA/TPA (7,12-dimethylbenz(a)anthracene and 12-*O*-tetradecanoylphorbol-13-acetate) chemical carcinogenesis protocol in which DMBA induces H-Ras mutations and TPA causes chronic inflammation, promoting tumour development^27^. Irradiated WT mice were reconstituted with WT (control) or TLR-5^−/−^ BM and topically treated with DMBA. Mice subsequently received repeated applications of TPA with or without prior wounding (Fig. 5a). Wound closure was accelerated in TLR-5^−/−^ BM chimeras treated with TPA (Supplementary Fig. 4a).

Mice treated with DMBA, but not TPA, and subsequently wounded, did not develop tumours (data not shown). Control chimeras that were wounded before TPA treatment developed tumours after 2 weeks of promotion, which is significantly faster than mice treated with TPA only (Fig. 5). There was a substantial delay in the development of DMBA/TPA-induced tumours in wounded TLR-5^−/−^ BM chimeras relative to wounded WT chimeras (Fig. 5b). The tumour-protective effect of TLR-5^−/−^ BM was also apparent in non-wounded DMBA/TPA-treated mice, albeit less marked (Fig. 5c). TLR-5^−/−^ BM reconstitution did not reduce the final number of papillomas that formed (Supplementary Fig. 4b,c) but decreased tumour size considerably (Fig. 5d,e).

We conclude that TLR-5-mediated signalling is involved in tumour initiation in two different skin cancer models.

### Upregulation of HMGB1 in wound-induced tumours

To investigate the relevance of mouse wound-induced tumour formation to human skin cancer, we analysed SCCs from RDEB patients. RDEB is a rare skin blistering condition in which repetitive cycles of wounding and repair predispose the skin to the development of SCCs^6^.

HMGB1 is a nuclear danger-associated molecular pattern that is passively released from necrotic cells and actively secreted by inflammatory cells^28^,^29^. HMGB1 is upregulated in RDEB patients^30^,^31^ and HMGB1 serum levels correlate with RDEB disease severity^30^. HMGB1 is also upregulated in a mouse RDEB model and mediates recruitment of BM-derived cells in injured tissue^31^. Furthermore, HMGB1 is induced in epithelial cells upon exposure to flagellin^32^. We therefore investigated HMGB1 as a candidate biomarker linking human and mouse wound-associated skin cancer.

**Figure 6:**
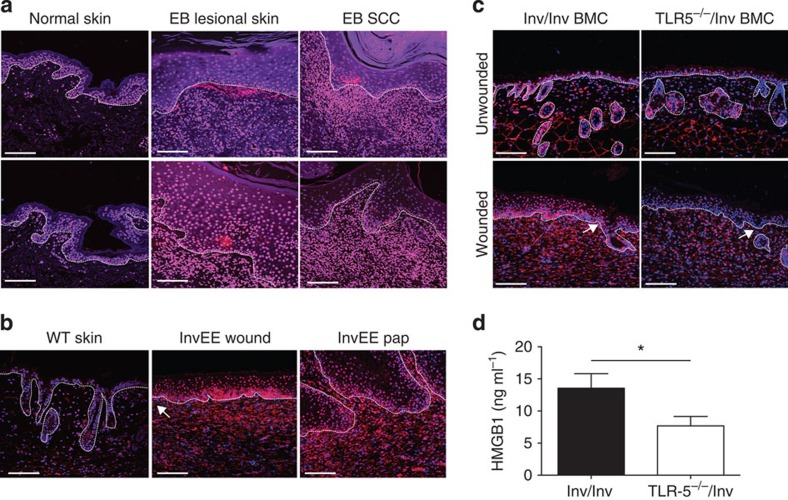
HMGB1 expression in RDEB and InvEE tumours (**a**–**c**) Paraffin sections were labelled with anti-HMGB1 (red) and counterstained with 4,6-diamidino-2-phenylindole (blue). Dotted line denotes basement membrane. Arrows denote wound edge. Scale bars, 200 μm. (**a**) Normal human skin, lesional RDEB skin and RDEB SCCs. (**b**) WT skin, InvEE healed wound bed (26 days post wounding) and wound-induced papilloma. (**c**) Unwounded and wounded InvEE/InvEE and InvEE/TLR5^−/−^ BM chimeras. (**d**) Serum levels of HMGB1 in non-tumour-bearing InvEE/InvEE and InvEE/TLR5^−/−^ BM chimeras at day 26 post wounding, assessed by ELISA (*n*=9 mice per condition; *0.01<*P*<0.05; Mann–Whitney *U*-test). Data are means±s.e.m.

In lesional skin from RDEB patients, HMGB1 was highly upregulated compared with normal human skin and there was strong immunoreactivity for HMGB1 in epidermis and dermis (Fig. 6a; *n*=6 patients per group). There was an even greater increase in HMGB1 immunoreactivity in RDEB SCCs (Fig. 6a; *n*=6 patients). Although HMGB1 was mainly nuclear in normal human skin, we observed cytoplasmic HMGB1 in lesional skin and SCCs from RDEB patients (Fig. 6a), which is indicative of HMGB1 secretion in these inflammatory conditions^29^. Consistent with these findings, HMGB1 was elevated in unwounded InvEE skin relative to WT (Fig. 6b,c) and further increased on wounding and in wound-induced papillomas (*n*=8; Fig. 6b,c). HMGB1 expression was significantly downregulated in skin of unwounded TLR-5^−/−^/Inv relative to Inv/Inv BM chimeras and the absence of TLR-5 prevented HMGB1 upregulation on wounding (Fig. 6c). The reduced immunolabelling in skin correlated with a reduction in serum HMGB1 levels in wounded mice (Fig. 6d).

We conclude that HMGB1 is upregulated in wound-associated mouse and human skin tumours and that HMGB1 levels are regulated by leukocytic TLR-5 signalling.

## Discussion

Host–microbe interactions are crucial for tissue homeostasis and evidence showing cancer-promoting effects of the commensal microbiome in various organs is accumulating^33^,^34^,^35^. Our studies establish, for the first time, a role for innate sensing of flagellated bacteria in wound-induced skin cancer. We demonstrate that TLR-5 ablation in BM-derived leukocytes reduced tumour incidence in mice and injection of the TLR-5 ligand flagellin induced papillomas in a TLR-5-dependent manner. Our analysis of RDEB tumours points to the relevance of our findings to human skin cancer. HMGB1, which is induced in epithelial cells in response to flagellin^32^, was strongly upregulated in wound-associated tumours in mice and RDEB patients. Ablation of TLR-5 in BM cells led to significantly reduced murine HMGB1 serum levels.

The skin microbiome composition is different across body surfaces^36^ and directs local immune responses^26^,^37^. The human microbiome project reference genome database reveals that intact skin contains flagellated bacterial species (based on the presence of *FliC* and *FliA* genes; http://www.hmpdacc.org/catalog/38). Consistent with this, a flagellated *E. coli* strain is present on InvEE skin^26^. Although most bacteria are kept at bay by the skin barrier, in a wound situation the moist and metabolite-rich environment can promote growth of opportunistic bacteria, including energetically expensive flagellated species^39^. Profiling the microbial configurations in chronic diabetic wounds and leg ulcers has indicated an increased representation of *E. coli*, *Pseuodomonas aeruginosa*, *Shigella* and other species comprising flagellated strains, whereas Staphylococci, a genus that does not comprise flagellates, are negatively correlated with ulcer duration^40^,^41^. Consistent with the observations in human skin, topical application of a broad-spectrum antibiotic was tumour protective in InvEE mice, whereas an antibiotic that targets Staphylocci was not. These observations lead us to propose that a key, and previously unrecognized, driver of wound-induced skin cancer is an increased exposure of leukocytes to flagellated bacteria.

Both of the mouse models in which we demonstrated a role for leukocytic TLR-5 sensing in wound-induced skin cancer are characterized by chronic inflammation. In InvEE mice, the inflammation results from epidermal expression of constitutively active MEK1 and in WT mice inflammation is induced by TPA treatment. TLR-5 ablation in BM does not abolish the inflammatory infiltrate in InvEE skin or TPA-treated WT skin. Our studies indicate that in the context of chronic inflammation, TLR-5 signalling in leukocytes can tip the balance between normal wound repair and tumour formation. The tumour-inhibitory effect of ablating TNFR or MyD88 in radiosensitive leukocytes in InvEE mice was greater than that of TLR-5 ablation (Figs 2a and 3a,d; 0.01<*P*<0.05; one-way analysis of variance), suggesting that additional components of the immune system that converge on NF-κB may contribute (Fig. 1d).

Our findings raise the possibility that the incidence of SCCs in non-healing ulcers and skin from RDEB patients could be reduced by systemic antibiotics treatment and the use of flagellin-specific targeting strategies in wound-induced malignancies might present an interesting clinical avenue.

## Methods

### Mice

InvEE mice^9^ were maintained on an F1 genetic background (CBA × C57Bl/6) and kept heterogeneous for the MEK1 transgene. Transgene negative littermates were used as WT controls and in DMBA/TPA experiments. All donor mice in the BM transplantation experiments were on a C57/Bl6 genetic background. TLR-5^−/−^ and TNFR-1/-2^−/−^ mice were purchased from Jackson Laboratories. TLR-2/-4^−/−^ mice were obtained from Simon Clare, IL-1R1^−/−^ mice from Nancy Rothwell, TRIF^−/−^ mice from Frederic Geissmann, TLR-7/-8^−/−^ mice from Lena Alexapoulou, MyD88^−/−^ mice from Caetano Reis e Sousa and TLR-9^−/−^ mice from Kinya Otsu. Animal procedures were subject to local ethical approval and performed under a UK Government Home Office Licence. Sample sizes were determined on the basis of prior power calculations. No mice died as a direct result of wounding or tumour formation. Mice that died as a result of myeloablation were excluded from analysis.

### Wounding and BM reconstitution

Full-thickness wounds were made on the back skin by using 2, 4, 5, 6 or 8 mm^2^ punch biopsy needles (Stiefel Instruments) under analgesia and general anaesthesia in 8- to 16-week-old age- and sex-matched InvEE and control littermates. Statistical power was calculated using the resource equation and animals were randomly assigned to treatment groups. No animals were excluded from any experiment. Tumour formation and size were measured by two independent researchers, who were blinded to group allocations.

In BM transplantation experiments^8^, 8- to 16-week-old female recipient mice were treated with acidified water at least 10 days before irradiation. Allogenic BM transplants were performed 24 h after myeloablative total body irradiation^42^ (two times 5 Gy, separated by 3 h) of InvEE mice. Donor BM was isolated from the tibia and femur of male mice. BM reconstitution was performed by intravenous injection of 5 × 10^6^ BM cells in 200 μl of PBS 24 h after irradiation. Chimerism was confirmed using Y-chromosome *in situ* hybridization (Cytocell, probe AMPOYR) on spleens of reconstituted mice^8^. Mice were wounded 4 to 6 weeks post BM reconstitution and papilloma formation was monitored for 50 to 60 days post wounding. Each InvEE BM reconstitution experiment was repeated two to three times.

### Treatment with antibiotics and flagellin

Mice were treated with the broad-spectrum antibiotic enrofloxacin by oral administration (5 ml Baytril per liter drinking water) starting 8 days before wounding and continuing until the end of the experiment. For topical applications, shaved back skin of mice was treated daily with 200 μl of vehicle (acetone) or antibiotics in acetone starting 8 days before wounding. Antibiotics used were 0.25% enrofloxacin or 0.2 mg ml^−1^ methicillin. Skin swabs were taken on day 10 post wounding and plated on lysogeny broth (LB) and blood agar plates to verify bacterial depletion. 16S rRNA quantification was performed on skin biopsies taken at the time of wounding.

1 or 4 μg flagellin (high-purity flagellin, EnzoLifeSciences) was injected once intradermally in back skin in 100 μl PBS; control mice were injected with 100 μl PBS. Flagellin was applied once topically to 8 mm^2^ full-thickness skin wounds at time of wounding.

### Quantitative PCR

Epidermis was separated from the underlying dermis by scraping skin after incubation at 60 °C for 7 s in RNase-free water. Isolated epidermis was homogenized in lysis buffer using ceramic beads. RNA extraction was performed according to the manufacturer’s instructions using a QIAGEN RNeasy Kit (Qiagen). Complementary DNA was generated using reverse transcriptase III (Invitrogen). Quantitative real-time PCR reactions were set up using gene-specific primer sets (see Supplementary Table 1) and reactions were performed on a 7900HT real-time PCR machine (Applied Biosystems) on biological triplicates.

### Microbiota quantification

Bacterial load was quantified in skin biopsy samples (8 mm^2^) and in faeces collected from mice 8 days after antibiotic treatment. Samples were digested with Ready-Lyse Lysozyme Solution (Epidentre Biotechnologies) in lysis buffer (20 mM Tris, pH 8.0, 2 mM EDTA, 1.2% Triton X-100, DNA-free water). Samples were homogenized using 5 mm Stainless Steel Beads (Qiagen) in a Precellys homogenizer twice at 6,000 r.p.m. for 50 s. DNA was extracted using a Purelink Genomic DNA kit (Invitrogen). For 16S rRNA quantification, the following primers were used: 5′-AGAGTTTGATCCTGGCTCAG-3′ and 5′-CTGCTGCCTCCCGTAGGAGT-3′. Data for skin bacterial load were normalized against the relative mouse genomic DNA amplicon quantification. Primers used were 5′-TTAGCAGTTTGGCACAGCTAGG-3′ and 5′-CTAGGTTGGCAAGGAATTGTGG-3′.

### ELISA

Blood was collected by cardiac puncture and left to clot for 2 h. Serum was collected after centrifugation for 20 min at 4 °C. TNF-α, IL-6 and TSLP levels were quantified using Quantikine ELISA kits (R&D systems). HMGB1 serum levels were measured by ELISA (Shino-test Corporation).

### DMBA/TPA two-stage carcinogenesis

Chemical carcinogenesis experiments were performed as previously described^27^. In brief, back skin of mice was shaved and treated once with 100 nmol (25 μg) DMBA in 200 μl acetone 5 weeks after BM reconstitution. Seven days later, mice were either wounded with an 8 mm^2^ full-thickness skin biopsy punch or treated with 6 nmol (3.7 μg) TPA. After 2 days, all mice were treated three times weekly with TPA for 15 weeks. Tumour incidence and burden were assessed once a week by two independent researchers who were blinded to the group allocations.

### Immunofluorescence staining

Frozen sections were fixed in acetone/methanol (1:1) or 4% paraformaldehyde/PBS pH 7.4 and blocked with 2% BSA, 0.02% fish skin gelatin and 10% goat serum for 1 h in PBS at room temperature. Paraffin sections were subjected to heat-mediated antigen retrieval (citrate buffer, pH6). When fluorochrome-conjugated primary antibodies were used, sections were incubated overnight at 4 °C in antibody solution containing 4',6-diamidino-2-phenylindole, washed in PBS and then mounted using the DAKO mounting reagent (DAKO). In the case of the unconjugated primary antibodies (HMGB1, Abcam, ab79823; anti-*Escherichia coli* antibody, DAKO, B0357), incubation was overnight at 4 °C followed by 2 h incubation at room temperature in secondary antibody (AlexaFluor 555 goat-anti-rabbit) containing 4,6-diamidino-2-phenylindole. Alexafluor 647-, 660- or 488-conjugated antibodies to CD3 (BioLegend, clone 17.A2), CD4 (eBioscience, clone RM4-5), F4/80 (eBioscience, clone BM8), CD19 (eBioscience, eFluor 660), CD11b (eBioscience, clone M1/70), CD11c (Cambridge Bioscience), γδ TCR (BD Pharmingen, clone GL3), NK1.1 (BioLegend, clone PK136) and CD207 (eBioscience, eFluor 660) were used.

### Immunohistochemistry

Dewaxed paraffin sections were subjected to heat-mediated antigen retrieval (citrate buffer, pH6). Mast cells were quantified by counting toluidine blue-stained cells on paraffin skin sections. CD45 (BD Pharmingen, clone 30-F11) antibody was used on paraffin sections at 1:100 dilution.

Quantification of immune cell infiltration was performed within the wound and adjacent to the wound (maximum distance from wound/pap: 4 mm) or in tumour stroma.

### Human samples

All human samples were collected after informed, written consent and in accordance with Helsinki guidelines, in compliance with the UK Human Tissue Act. The study protocol was approved by the Universities of Monterrey and New South Wales.

### Statistics

Statistical analyses were performed using GraphPad Prism v6. Specific tests included Fisher’s exact tests, one or two-way analysis of variance, unpaired *t*-tests and Mann–Whitney tests. *0.01<*P*<0.05, **0.01<*P*<0.001, ***0.0001<*P*<0.001, ****P<0.0001.

## Additional information

**How to cite this article:** Hoste, E. *et al.* Innate sensing of microbial products promotes wound-induced skin cancer. *Nat. Commun.* 6:5932 doi: 10.1038/ncomms6932 (2015).

## Supplementary Material

Supplementary Information Supplementary Figures 1-3 and Supplementary Table 1
