# Supplementary material for: Innate sensing of microbial products promotes wound-induced skin cancer
Source: Nat Commun. 2015 Jan 9;6:5932. doi: 10.1038/ncomms6932 (PMC4338544; doi:10.1038/ncomms6932)
Supplement: Supplementary Information — Supplementary Figures 1-3 and Supplementary Table 1 [file ncomms6932-s1.pdf]

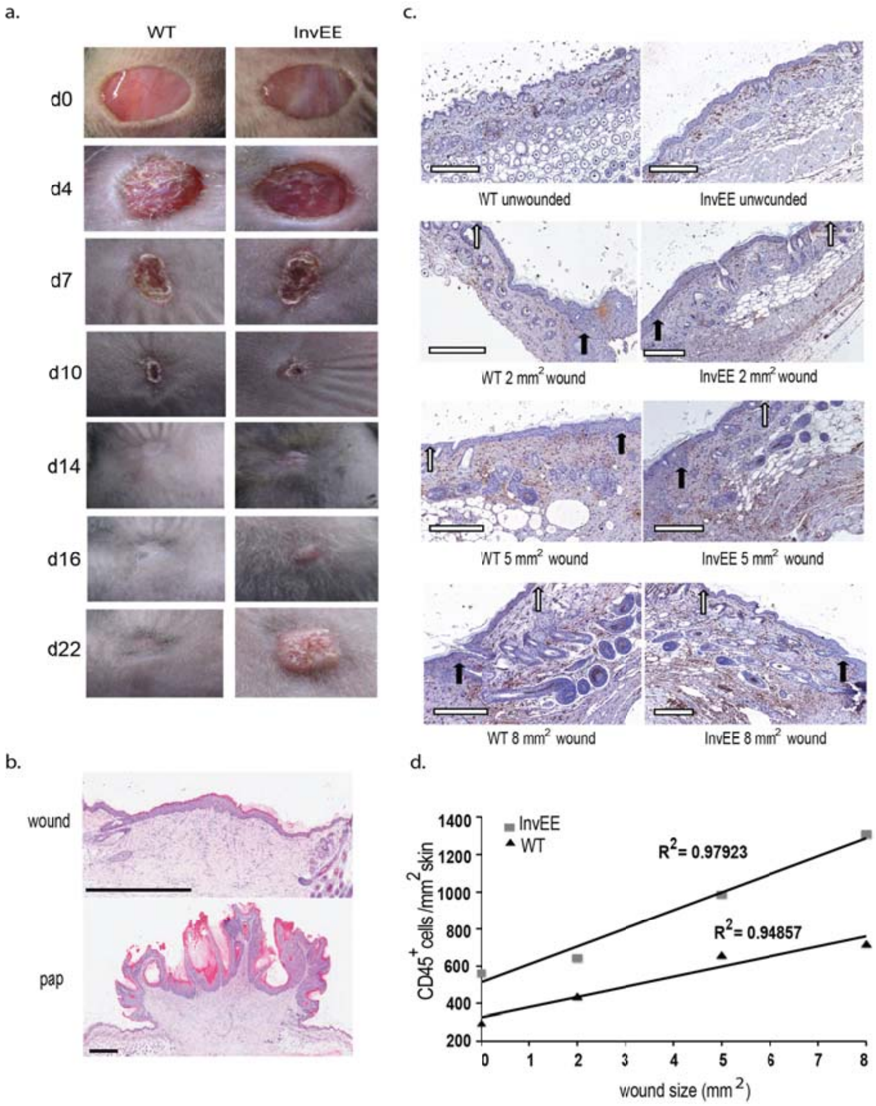

Supplementary Figure 1: Effect of wound size on inflammatory infiltrate (a) Macroscopic images of WT and InvEE 8 mm^2 wounds at day 0, 4, 7, 10, 14, 16 and 22 post-wounding. **(b)** Haematoxylin-eosin stained sections of a healed wound and papilloma (pap) 30 days after wounding. Scale bars: 1 mm. **(c)** Representative images of CD45 stained skin during healing of different sized wounds. CD45+ cells were quantified in the region between the arrows. Black arrows indicate wound edges. Scale bars: 300 μm. **(d)** Correlation between wound size and number of CD45+ leukocytes in wound beds of healed InvEE and WT skin. Mice were harvested at time of wound closure (n≥ 4 mice per condition, at least 3 microscopic fields were quantified per mouse).

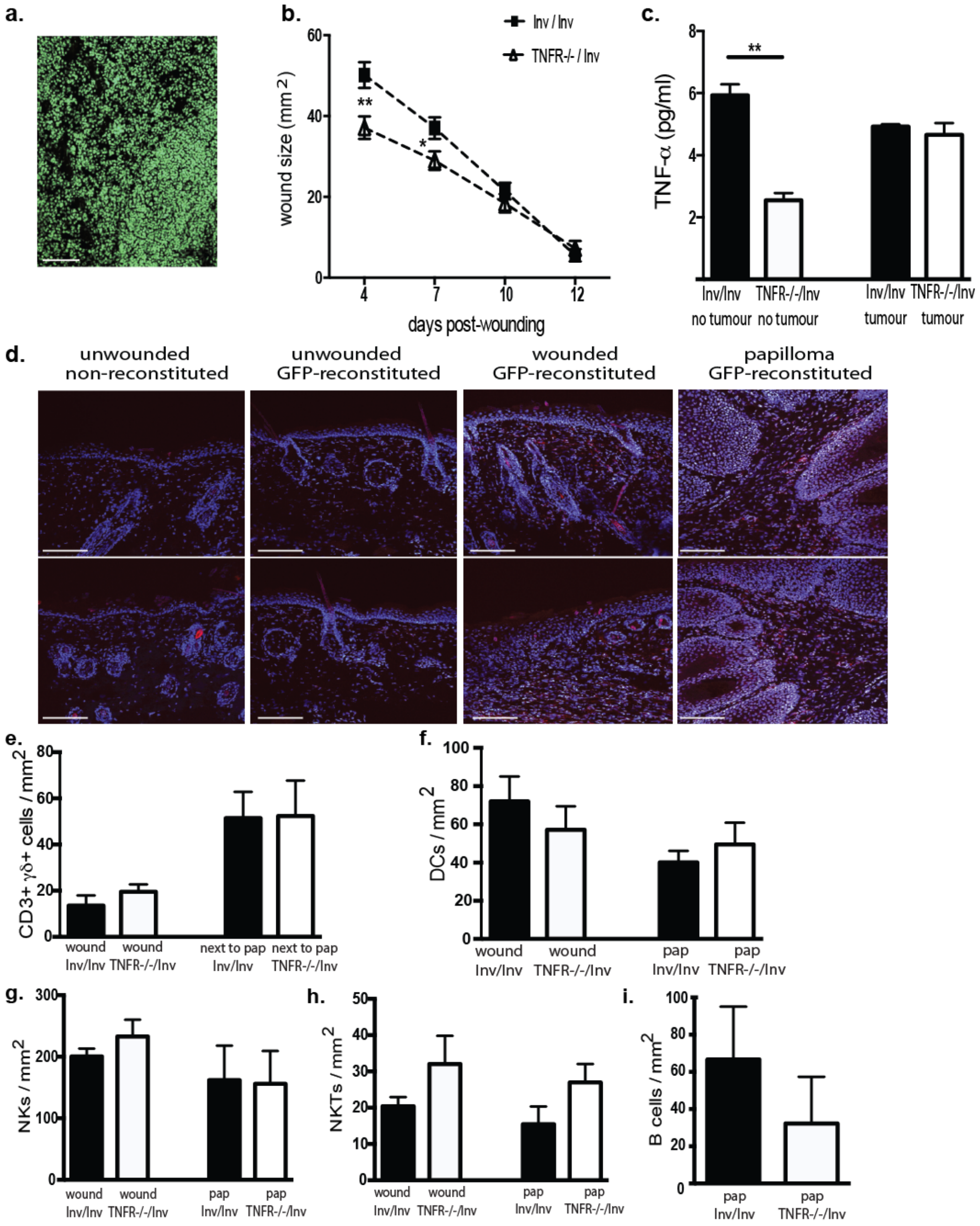

Supplementary Figure 2: Bone marrow reconstitution and immune cell infiltration (a) Y-chromosome in situ hybridization (green) on spleen of female mouse reconstituted with male BM collected 6 weeks after reconstitution. Scale bar, 100μm. **(b)** Wound healing dynamics in TNFR$^{-/-}$ and control BM chimeras (n=12 mice per condition; * 0.01<p<0.05, ** 0.01<p<0.001; unpaired t-test). **(c)** Serum levels of TNF-α in TNFR$^{-/-}$ and control BM chimeras with or without wound-induced papillomas (n=4 mice per condition; ** 0.01<p<0.001; Mann-Whitney U-test). **(d)** Labeling of F4/80 (red)

macrophages in non-reconstituted irradiated mice (24h after irradiation) or mice reconstituted with BM from mice that express eGFP (green) under control of the β-actin CMV promoter. DAPI nuclear counterstain is shown in blue. Scale bars, 200 μm. **(e)** Quantification of γδ T-cells (double labeled with anti-γδ TCR and anti-CD3 antibodies) in wound beds or skin adjacent to papillomas. **(f)** Quantification of dendritic cells (double labeled with anti-CD11c and anti-CD207 antibodies) in wound beds and tumour stroma. **(g, h)** Quantification of NK (labeled with anti-NK1.1) and NK T-cells (double labeled with anti-NK1.1 and anti-CD3 antibodies). **(i)** Quantification of B cells (labeled with anti-CD19 antibodies) in tumour stroma. (**e-i**) Quantification was performed on ≥ 3 mice per condition, and at least 3 fields were counted per section analysed. Graphs represent means ± SEM.

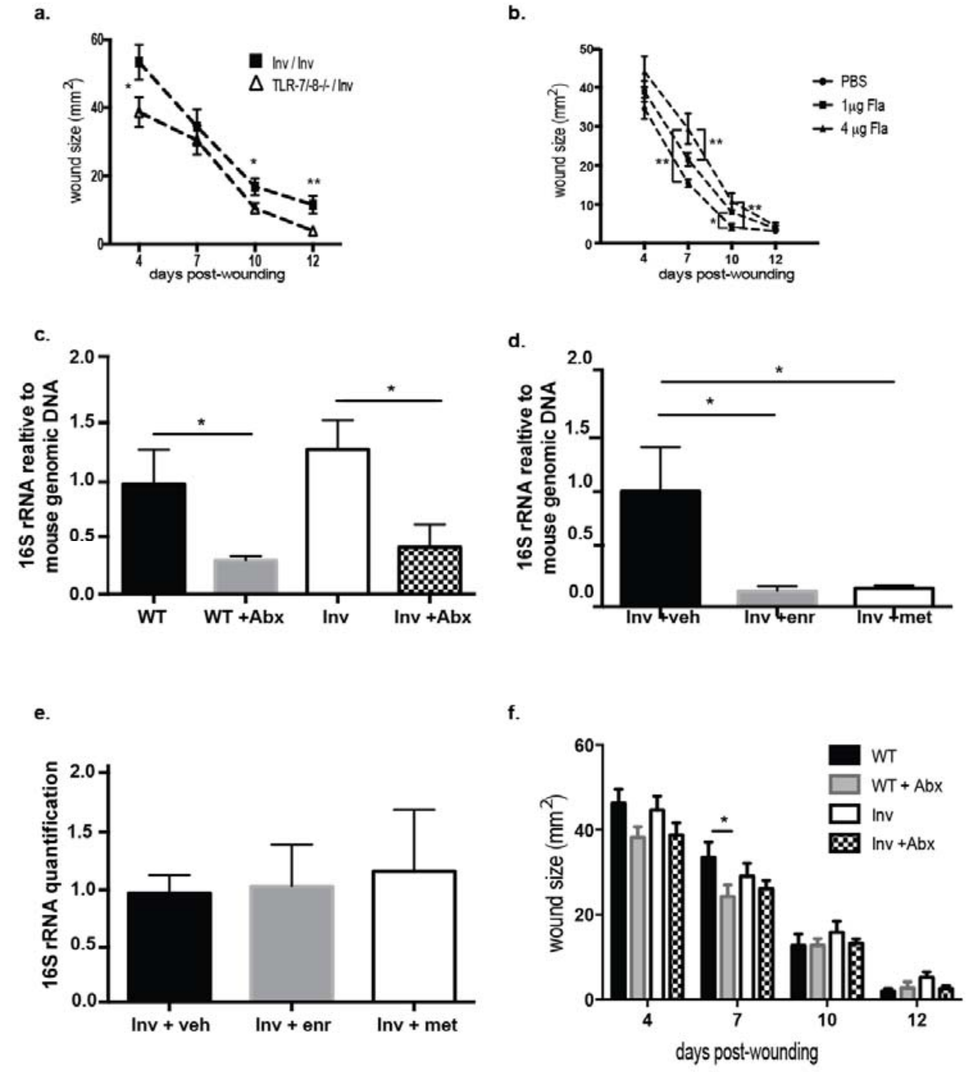

Supplementary Figure 3: Wound healing dynamics and bacterial load (a) Wound healing dynamics in TLR-7/-8$^{-/-}$ and control BM chimeras (n=12 per condition; * 0.01<p<0.05, ** 0.01<p<0.001; unpaired t-test). **(b)** Wound healing dynamics in InvEE wounds treated with PBS or flagellin (n≥ 8 mice per condition; ** 0.01<p<0.001; unpaired t-test). **(c)** 16S rRNA Q-PCR normalized to mouse genomic DNA of skin biopsies collected at time of wounding. Mice had been pre-treated with or without orally administered antibiotics (Abx) for 8 consecutive days (n>4 mice per condition; * 0.01<p<0.05; Mann-Whitney U-test). **(d, e)** Mice had been topically treated with (enr, met) or without (veh) antibiotics for 8 consecutive days (n>4 mice per condition; * 0.01<p<0.05; Mann-Whitney U-test). Enr: enrofloxacin; met: methicillin. **(d)** 16S rRNA Q-PCR of skin biopsies normalized to mouse genomic DNA. **(e)** 16S rRNA Q-PCR of faeces collected at the time of

wounding. **(f)** Wound healing dynamics in mice treated with or without antibiotics in the drinking water (n>9 per condition; * 0.01<p<0.05; Mann-Whitney U-test). All data are means ± SEM.

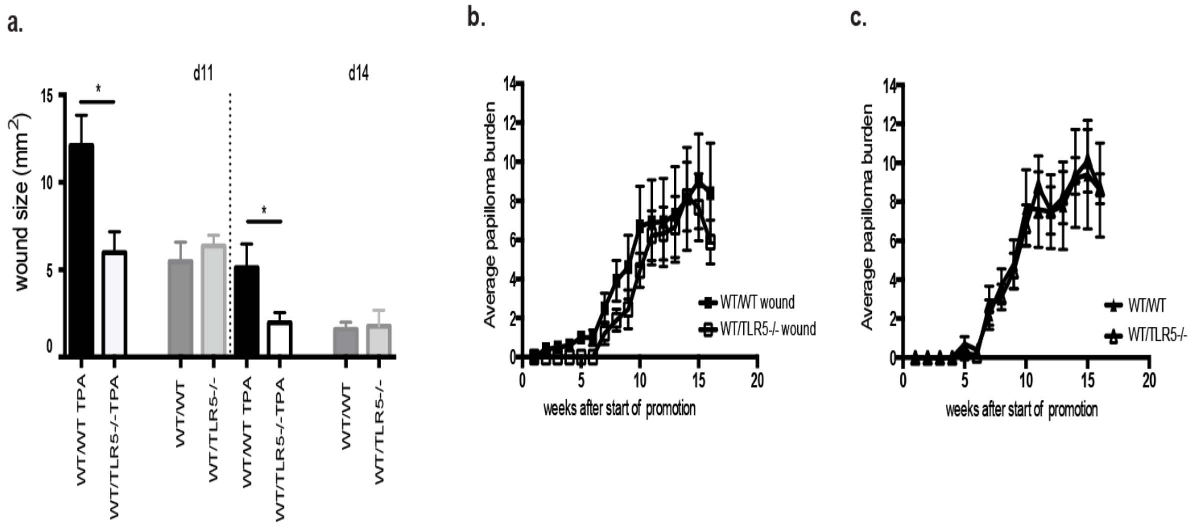

Supplementary Figure 4: Role of TLR-5 in chemical carcinogenesis (a) Wound area in wild-type mice reconstituted with TLR-$5^{-/-}$ or control (WT) BM at day 11 and 14 after wounding. Mice that were treated with DMBA and TPA are compared with mice that were treated with DMBA only (n>8 mice per condition; * 0.01<p<0.05; unpaired t-test). Data are means ± SEM. **(b)** Number of papillomas per mouse in TLR-$5^{-/-}$ and control BM chimeras wounded and treated with DMBA/TPA (n≥ 9 mice per condition). Data are means ± SEM. **(c)** Number of papillomas per mouse in TLR-$5^{-/-}$ and control BM chimeras treated with DMBA/TPA without wounding (n≥ 9 mice per condition). Data are means ± SEM.

Supplementary Table 1: Quantitative PCR primer sets

Gene	Forward primer	Reverse primer
IL-1α	GGGCTGGTCTTCTCCTTGAG	TTGGTTAAATGACCTGCAACA
IL-1β	GCTTCCAAACCTTTGACCTG	CTGTTGTTTCCCAGGAAGAC
TSLP	TCTCAGGAGCCTCTTCATCCT	CTCACAGTCCTCGATTTGCTC
TGF-α	TATCACCTGTGTGCTGATCCA	CAAGCAGTCCTTCCCTTCAG
TGF-β1	ATACGTCAGACATTCGGGAAG	GACGTCAAAAGACAGCCACTC
MyD88	GTAGACAGGACGGCATCAGAG	CTATACCAACCCTTGCACCAA
Bcl-XL	CTGCTCACTTACTGGGTCTGC	AAGAGGCGGATGAAACAATTC
JNK	ATGGGTCTGATTCTGAAATGG	CAGAAGCAAACGTGACAACAA
IRF-3	GATGGCTGACTTTGGCATCT	GGTCCTTGCTATTGTCAGCAG
NF-κB	GCTGTCACTATCCCGGAGTTC	TCTGGGGGTACCATCAAAGAG
S100A8	ATCCTTTGTCAGCTCCGTCTT	TGTAGAGGGCATGGTGATTTC
S100A9	GCTGATTGTCCTGGTTTGTGT	GACAAATGGTGGAAGCACAGT
IL-22	AGACAGGTTCCAGCCCTACAT	TCTTCTGGATGTTCTGGTCGT
Iκbα	GTTGACATCAGCACCCAAAGT	ACCCCTCTACATCTTGCCTGT
TNFAIP3	CTTCTGAGGATGTTGCTGAGG	CAGGAATTTGTGGAAACAGGA
MCP-1	AGCTCTCTCTTCCTCCACCAC	TGCTGGTGATCCTCTTGTAGC
GAPDH	AACATCAAATGGGGTGAGGCC	GTTGTCATGGATGACCTTGGC
